# Minor Effect of Antibiotic Pre-treatment on the Engraftment of Donor Microbiota in Fecal Transplantation in Mice

**DOI:** 10.3389/fmicb.2019.02685

**Published:** 2019-11-21

**Authors:** Tobias L. Freitag, Anna Hartikainen, Hanne Jouhten, Cecilia Sahl, Seppo Meri, Veli-Jukka Anttila, Eero Mattila, Perttu Arkkila, Jonna Jalanka, Reetta Satokari

**Affiliations:** ^1^Translational Immunology Research Program, Faculty of Medicine, University of Helsinki, Helsinki, Finland; ^2^Department of Bacteriology and Immunology, Faculty of Medicine, University of Helsinki, Helsinki, Finland; ^3^Human Microbiome Research Program, Faculty of Medicine, University of Helsinki, Helsinki, Finland; ^4^Department of Infectious Disease, Helsinki University Central Hospital, Helsinki, Finland; ^5^Department of Gastroenterology, Helsinki University Central Hospital, Helsinki, Finland

**Keywords:** microbiota, fecal transfer, antibiotics, dysbiosis, *bifidobacteria*, microbiome

## Abstract

Fecal microbiota transplantation (FMT) is an effective therapy for recurrent *Clostridioides difficile* infection (rCDI) and is also considered a potential treatment for a wide range of intestinal and systemic diseases. FMT corrects the microbial dysbiosis associated with rCDI, and the engraftment of donor microbiota is likely to play a key role in treatment efficacy. For disease indications other than rCDI, FMT treatment efficacy has been moderate. This may be partly due to stronger resilience of resident host microbiota in patients who do not suffer from rCDI. In rCDI, patients typically have undergone several antibiotic treatments prior to FMT, depleting the microbiota. In this study, we addressed the effect of broad-spectrum antibiotics (Ab) as a pre-treatment to FMT on the engraftment of donor microbiota in recipients. We conducted a pre-clinical study of FMT between two healthy mouse strains, Balb/c as donors and C57BL/6 as recipients, to perform FMT within the same species and to mimic interindividual FMT between human donors and patients. Microbiota composition was assessed with high-throughput 16S rDNA amplicon sequencing. The microbiota of Balb/c and C57BL/6 mice differed significantly, which allowed for the assessment of microbiota transplantation from the donor strain to the recipient. Our results showed that Ab-treatment depleted microbiota in C57BL/6 recipient mice prior to FMT. The diversity of microbiota did not recover spontaneously to baseline levels during 8 weeks after Ab-treatment, but was restored already at 2 weeks in mice receiving FMT. Interestingly, pre-treatment with antibiotics prior to FMT did not increase the overall similarity of the recipient’s microbiota to that of the donor’s, as compared with mice receiving FMT without Ab-treatment. Pre-treatment with Ab improved the establishment of only a few donor-derived taxa, such as *Bifidobacterium*, in the recipients, thus having a minor effect on the engraftment of donor microbiota in FMT. In conclusion, pre-treatment with broad-spectrum antibiotics did not improve the overall engraftment of donor microbiota, but did improve the engraftment of specific taxa. These results may inform future therapeutic studies of FMT.

## Introduction

Fecal microbiota transplantation is an effective and curative treatment for rCDI (Mattila et al., 2012; van Nood et al., 2013; Quraishi et al., 2017), also eliminating rCDI-associated dysbiosis (Fuentes et al., 2014; Jalanka et al., 2016). Randomized clinical trials using FMT as a treatment for other intestinal diseases, such as UC (Narula et al., 2017) or IBS (Xu et al., 2019), have, however, had moderate or little success. One difference in the treatment regimens utilizing FMT for rCDI compared with other diseases is the use of antibiotics. Usually, rCDI patients are treated with antibiotics (e.g., vancomycin or metronidazole) prior to FMT, but antibiotics are rarely used before FMT for other indications. Generally, patients will undergo bowel lavage, provided that FMT is administered via colonoscopy.

Antibiotics are known to modulate the composition of the intestinal microbiota in both quantitative and qualitative terms (Dethlefsen and Relman, 2011; Ianiro et al., 2016), and their therapeutic efficacy has been demonstrated in non-infectious diseases such as IBS and hepatic encephalopathy (Ianiro et al., 2016). In addition to antibiotics, bowel lavage will reduce the number of intestinal bacteria and affect the microbial ecosystem temporarily (Morotomi et al., 1989; Jalanka et al., 2015). Although recent studies have shown that donor microbiota and specific donor-derived strains colonize the recipient after FMT (Jalanka et al., 2016; Li et al., 2016; Allegretti et al., 2019), it remains unclear whether donor strain engraftment is a prerequisite for the therapeutic efficacy of FMT in rCDI. In published studies of FMT for both rCDI and UC, similarity between donor and recipient microbiota profiles was higher in treatment responders (Moayyedi et al., 2015; Rossen et al., 2015; Staley et al., 2016), arguing for a critical role of the donor strain engraftment in treatment efficacy in patients undergoing FMT.

It has been hypothesized that antibiotic pre-conditioning may lead to a more effective transfer of the donor microbiota to the recipient, resulting in a higher similarity between donor and recipient microbiota profiles after FMT. If this were true, antibiotic pre-conditioning might lead to better treatment outcomes in patients undergoing FMT. Few previous experimental studies have also addressed the contribution of antibiotic pre-treatment before FMT to donor microbiota engraftment efficacy. Three studies that analyzed the effect of antibiotics before transplantation of microbiota from human feces into mice, thus addressing the interspecies transfer, produced divergent findings, suggesting that broad-spectrum antibiotics may support microbiota engraftment across the species barrier (Wos-Oxley et al., 2012; Ji et al., 2017; Staley et al., 2017). Only two studies analyzed the effects of broad-spectrum antibiotics before FMT between different strains of the same mammalian species. In rats, antibiotic pre-treatment did not improve the engraftment of donor’s microbiota (Manichanh et al., 2010), whereas in juvenile mice antibiotics in combination with bowel lavage enhanced donor microbiota engraftment (Le Roy et al., 2018). However, this effect appeared to depend mainly on bowel lavage.

To further test the hypothesis that antibiotic pre-treatment before the administration of FMT will enhance microbiota engraftment, we performed an experimental study of FMT between adult mice from two different inbred strains, Balb/c as the donor and C57BL/6 as the recipient, mimicking interindividual differences between human donors and patients. We compared microbiota changes in recipient mice pre-treated with a cocktail of broad-spectrum antibiotics (ciprofloxacin, metronidazole, and vancomycin) and receiving FMT after bowel lavage with the changes in recipient mice treated with bowel lavage and FMT, or FMT alone. These results were compared with those of a group of recipient mice receiving Ab-treatment and sham FMT, to analyze the compositional changes of recipient microbiota introduced by antibiotics.

## Materials and Methods

### Mice and Treatments

Four adult Balb/c female mice (FMT donors) were purchased from Envigo (Madison, WI, United States), maintained under specific pathogen-free conditions in ventilated/autoclaved cages, and fed sterilized chow (Teklad Global 16% Rodent Diet 2916C, Envigo). A colony of C57BL/6 mice (FMT recipients), originally purchased from The Jackson Laboratory (Bar Harbor, ME, United States), were maintained in the same facility and under the same conditions, but in strict isolation from the Balb/c mice. One week after arrival of the Balb/c mice at the facility, cages were changed in the evening, and stool pellets were collected from all four stool donors next morning. The stool material was stored as described previously (Satokari et al., 2015). Briefly, 500 mg of stool was suspended in 5 ml of sterile PBS, filtered through an autoclaved metal sieve, and 5 ml of suspensions containing 10% glycerol were stored at −80°C until use. Donor mice were sacrificed after stool sample collection, and coecum and colon segments were frozen at −80°C. Two samples of pooled donor feces were used for microbiota analysis.

**FIGURE 1:**
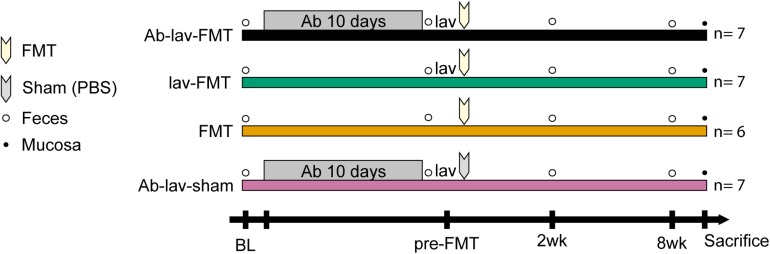
Study design showing the treatment groups of recipient mice. After sampling of feces at baseline (recipients before treatments, BL) from four groups of mice, two groups of mice were pre-treated for 10 days with an antibiotic cocktail (Ab), consisting of 660 mg/l ciprofloxacin, 1 g/l metronidazole, and 500 mg/l vancomycin in drinking water. Two groups of mice received fecal microbiota transplantation (FMT) after bowel lavage (lav), one group received FMT without lav, and one groups received sham FMT with phosphate-buffered saline (PBS) after lav. The time points of fecal (empty bullets) or mucosal (full bullets) samplings are indicated.

Four groups of female C57BL/6 mice, maintained under the conditions specified above, were matched from 7 litters (3–5 months old; *n* = 6–7), following collection of stool samples from all individuals on day 1 (baseline, BL; collection as above, Figure 1). On the same day, the drinking water for the two antibiotic pre-treated groups was supplemented with 660 mg/l ciprofloxacin (Yliopiston Apteekki, Helsinki, Finland), 1 g/l metronidazole (Sigma-Aldrich, St. Louis, MO, United States), and 500 mg/l vancomycin (Orion Pharma, Espoo, Finland). On day 10, antibiotic treatment was stopped. Stool samples were collected from all individuals on day 11 (time point pre-FMT). On the same day, three of the groups were treated with 200 μl of colonsteril by oral gavage, containing 12 mg of macrogol 4000, prepared according to the manufacturer’s instructions (Orion Pharma, Espoo, Finland). Mice from all of the groups received one injection intraperitoneally of 2.5 mg of ranitidine hydrochloride in 100 μl volume (GlaxoSmithKline, Zeist, Netherlands). Five hours later, 200 μl of pooled, thawed stool suspension (equal to 20 mg of stool) from Balb/c donor mice was administered by oral gavage to three groups of mice, while the remaining sham-FMT group received 200 μl of 10% glycerol in PBS. On days 25 (time point 2 weeks) and 71 (time point 8 weeks), stool samples were collected from all individuals and stored at −80°C for microbiota analysis. On day 72, mice were injected with ketamine/xylazine, and exsanguinated by retroorbital bleeding. Coecum and colon segments were collected and frozen at −80°C for microbiota analysis. All animal procedures were approved by the Southern Finnish State Administrative Agency (ESAVI/1286/04.10.07/2016).

### Fecal Sample DNA Extraction

A previously described, a validated method for fecal sample microbial DNA extraction was used (Salonen et al., 2010; Santiago et al., 2014). Repeated bead beating was used as a pre-treatment, followed by high-throughput DNA extraction using KingFisher Flex 96 (Thermo Fisher Scientific, Vantaa, Finland). Extracted DNA was purified with QIAamp DNA Mini-kit (Qiagen, Hilden, Germany), and concentration measured using the Quant-iT^TM^ PicoGreen^TM^ dsDNA Assay Kit (Invitrogen^TM^, Eugene, OR, United States).

### Biopsy DNA Extraction

DNA from the biopsy samples was extracted using a previously described method (Kalliomäki et al., 2012). In short, enzymatic lysis and repeated bead beating were used to disrupt microbial cells. DNA was extracted using the Qiagen DNeasy mini-spin column (Qiagen, Hilden, Germany), according to the manufacturer’s instructions for Gram-positive bacteria. DNA concentration was measured using the Quant-iT^TM^ PicoGreen^TM^ dsDNA Assay Kit (Invitrogen^TM^, Eugene, OR, United States).

### Microbial DNA Compositional Analysis

Microbial composition in fecal samples was analyzed by sequencing the hypervariable V3–V4 region of the 16S rRNA gene with Illumina MiSeq (Klindworth et al., 2013). For the analysis of mucosal microbiota, full-length 16S rRNA PCR was conducted first to enrich the microbial DNA in biopsy DNA samples. Briefly, 1500 bp amplicon was generated by using the previously designed primers (Wang et al., 2002) and by using the following amplification conditions: one cycle of 95°C for 2 min, followed by 20 cycles of 95°C for 20 s, 55°C for 20 s, 72°C for 90 s, and a final extension cycle of 72°C for 5 min. PCR products were purified using the QIAquick^®^ PCR Purification Kit (Qiagen, Hilden, Germany) according to the manufacturer’s instructions. The purified PCR product was used as the template for 16S rRNA gene V3-V4 region sequencing similarly to the fecal samples by using the MiSeq platform. The analysis was successful for 102 of 108 fecal samples and for 36 of 54 biopsy samples. The data were deposited to the European Nucleotide Archive (PRJEB33286).

For processing and analyzing the reads, R package mare was used (Korpela, 2016). Forward reads trimmed to 150 nt were used for the analysis. This approach has been previously observed to produce highly reliable results when using artificial communities of known composition (Korpela, 2016). To exclude errors and chimeras, rare reads were eliminated by using a quality filter for minimal read abundance of 0.001%, and reads were chimera filtered. After pre-processing, the average read number was 49,607 per sample (ranging from 6,249 to 335,038, median 46,629). Taxonomic annotation was made by using the USEARCH (version 8.1.1756) and SILVA 16S rRNA reference database version 115 (Quast et al., 2013).

### Statistical Methods

For statistical analysis, R package mare was used (Korpela, 2016). It uses tools from R packages vegan (Oksanen et al., 2019), MASS (Venables and Ripley, 2002), and nlme (Pinheiro et al., 2019). Genus-level data were used for the statistical testing. To test differences in the bacterial abundance between study groups at certain time points, generalized linear mixed models using the function glm.nb from package MASS were used. If there was a failure in fulfilling the model assumptions, alternatively generalized least squares models were used. The read number for each sample was used as an offset. The obtained *p*-values were corrected using multiple testing with false discovery rate approach, and FDR-adjusted *p*-values (*q*-values) below 0.05 were considered to be significant. Principal co-ordinate analysis was used to visualize microbial composition at different time points and study groups. The function used utilizes R package vegan and Bray-Curtis dissimilarities. Moreover, to estimate the grouping effect on overall microbiota composition, adonis function (PERMANOVA) in R package vegan was used. Additionally, ANOVA followed by Tukey’s HSD *post hoc* test or Student’s *t*-test was used for evaluating statistical significance in normally distributed data. Normality of the data was tested by using the Shapiro-Wilk test. Similarity between the samples was calculated with Spearman’s correlation (ρ). OriginPro 2018 (version b9.5.5.409 Academic) and CorelDRAW (version 20.1.0.708) were used for visualizing the data.

## Results

The main aim of this study was to analyze the effect of antibiotic pre-treatment on microbiota engraftment after FMT. The study design is depicted in Figure 1. Briefly, we compared the microbial changes of the recipient mice pre-treated with antibiotics and receiving FMT after bowel lavage with the three different control groups. These included mice that had also received antibiotic pre-treatment and sham FMT after the lavage, in addition to a group of mice treated with either bowel lavage and FMT or FMT alone.

### Fecal Microbial Populations in Donors and Recipients Differed at Baseline

**FIGURE 2:**
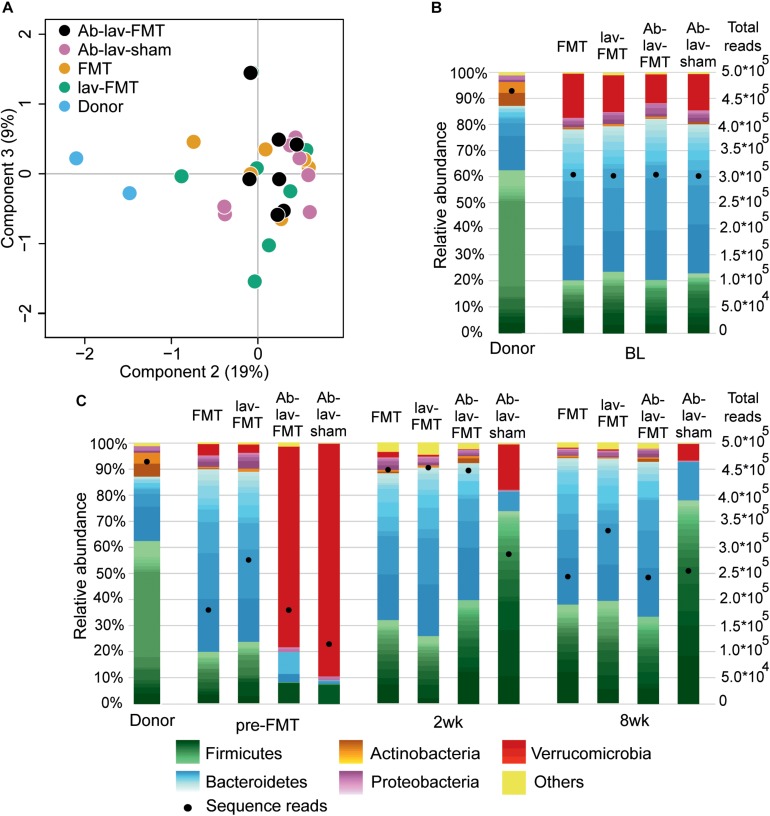
**(A)** PCoA of microbiota profiles at bacterial genus level from the study groups at baseline. **(B)** Fecal microbiota composition in donor and recipient mice at baseline. Relative abundance scaled to 100%, and number of sequence reads marked with bullets. Within phyla, individual genera are shown with different shades, and the lightest color shows the combined abundance of the least abundant genera (<0.5% of total). **(C)** Microbiota composition of donor and recipient mice at other time points in the study.

At baseline, the fecal microbiota in Balb/c donors differed significantly from that in C57BL/6 recipients (Figures 2A,B and Supplementary Figure S1A). The differences between these mouse strains explained 18% (*p* = 0.002, PERMANOVA) of the microbial variation, and the microbiota similarity between the recipients and donors, measured with Spearman’s correlation, was on average 0.57 ± 0.03 (SD). At bacteria phylum level, the differences included higher levels of Firmicutes and Actinobacteria in donors (62.62 vs. 21.80%, and 9.20 vs. 0.83%, respectively, Figure 2B). Recipient mice had higher levels of Bacteroidetes and Verrucomicrobia (58.25 vs. 24.72%, and 14.11 vs. 0.04%, respectively). On average, there were 39.75 ± 2.22 genus-level taxa that had statistically different abundance (FDR-adjusted *p* < 0.05) between donors and recipients (Supplementary Table S1). The observed microbiota differences between the mouse strains allowed for the assessment of microbiota engraftment from the donor strain to the recipient based on microbiota profiling.

### Effect of Antibiotic Treatment on Microbiota Composition

**FIGURE 3:**
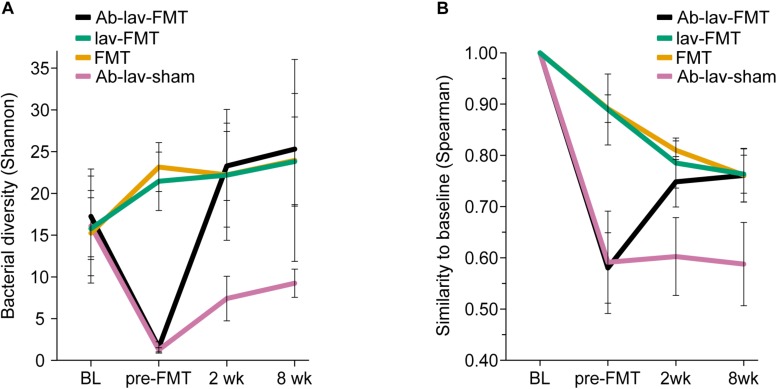
**(A)** Bacterial diversity in different treatment groups across all time points. **(B)** Similarity between the baseline sample and consecutive samples in all study groups across all time points.

We first assessed the effect of antibiotic treatment on the microbiota. In addition to the group that later received FMT after antibiotics pre-treatment (Ab-lav-FMT), a control group of mice that later received sham FMT was also pre-treated with antibiotics (Ab-lav-sham). The antibiotic treatment had a strong effect on microbiota, with 79% of the microbial variation between the study groups explained by the consumption of antibiotics (*p* = 0.01, PERMANOVA, Figure 2C and Supplementary Figure S1B) at time point pre-FMT. Microbial diversity was decreased significantly in the antibiotic-treated animals compared with the untreated animals (*p* = 9.96 × 10^–12^, ANOVA, Tukey’s *post hoc*, Figure 3A). The diversity did not recover to baseline levels in the Ab-lav-sham group during the follow-up period. This was reflected also in the similarity analysis, where the Ab-lav-sham group showed little similarity to their own baseline microbiota, both at 2 weeks (ρ = 0.60 ± 0.08, Figure 3B) and at 8 weeks (ρ = 0.59 ± 0.08, Figure 3B) after sham FMT.

The microbial composition of animals treated with antibiotics was significantly reduced after the antibiotic treatment (at time point pre-FMT), and only 44 of the 117 genera detected at baseline were present in over 0.01% abundance (Supplementary Table S2). Here, the most abundant genus-level taxa were *Verrucomicrobium* (84.78%), *Lactobacillus* (6.70%), uncultured member of Bacteroidetes (2.38%), and *Parabacteroides* (1.63%), representing on average 95.49% of all detected taxa (Supplementary Table S3).

### Effect of Antibiotic Pre-treatment on Microbiota Engraftment

Next, we studied the recovery of antibiotics-depleted microbiota by FMT, and the differences in donor microbiota engraftment between the animals that received and those that did not receive antibiotics before FMT. The dramatic decrease in microbial diversity caused by the antibiotics was restored by FMT (Figure 3A). The increase in diversity was detected already 2 weeks after the treatment in the Ab-lav-FMT group, and it was sustained until the end of the study (Figure 3A). Compared with the control groups, diversity of the Ab-lav-FMT group was similar to the mice that were treated with FMT but did not receive antibiotics (FMT and lav-FMT) (Figure 3A). However, in the Ab-lav-sham group, diversity remained low at 2 and 8 weeks after sham FMT.

**FIGURE 4:**
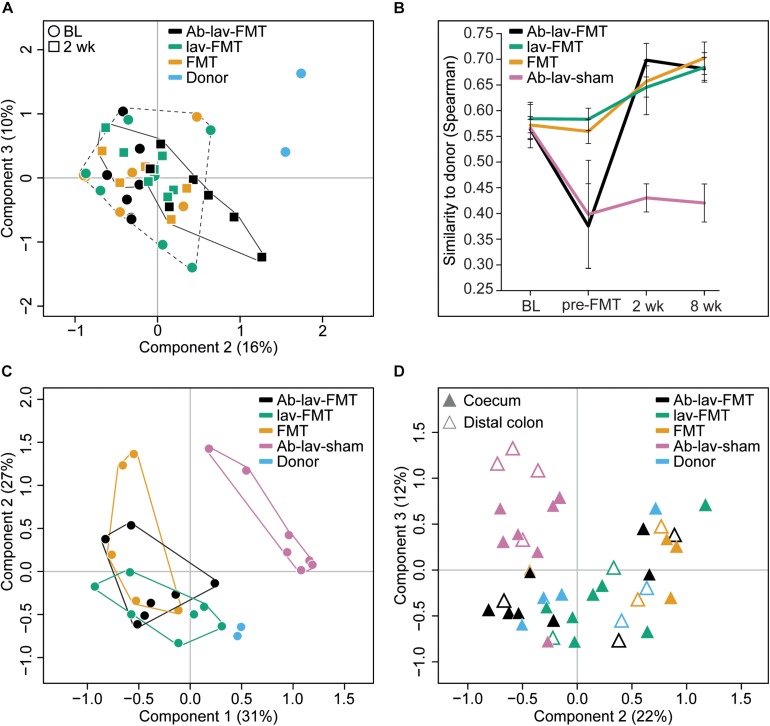
**(A)** PCoA of fecal microbiota composition: effects of antibiotic pre-treatment and study time point. Ab-lav-sham group is excluded to visualize the distribution of the FMT group samples in more detail. **(B)** Microbial similarity to donor for different groups throughout the study. Similarity measured with Spearman correlation. **(C)** PCoA of fecal microbiota composition at 8 weeks after (sham) FMT: significant separation of Ab-lav-sham group from the other groups (*p* = 0.001, PERMANOVA). **(D)** PCoA of mucosal microbiota composition at 8 weeks after (sham) FMT: significant effects of antibiotic treatment (*p* = 0.001, PERMANOVA) and biopsy location (*p* = 0.001, PERMANOVA).

When visualizing compositional dissimilarity with PCoA, the donor’s microbiota was found to cluster separately from that of FMT-treated animals. In addition, the samples from baseline and after FMT were mixed and did not show any significant shift toward the donor samples (Figure 4A and Supplementary Figure S1C). This observation was supported by the similarity analyses showing a higher correlation of the microbial composition in mice from all FMT groups with their own baseline (ρ = 0.77, ± 0.02, Figure 3B) than with donors (ρ = 0.68 ± 0.02, Figure 4B). In this study, the within-animal similarity in the C57BL/6 mice was 0.89 ± 0.05.

However, the similarity between donors and recipients was low at baseline (ρ = 0.57 ± 0.03), and it increased significantly 2 weeks after FMT in the treated animals (Ab-lav-FMT: 19.21% increase, *p* = 2.73 × 10^–5^; lav-FMT: 9.44% increase, *p* = 0.001; FMT group: 12.89% increase, *p* = 0.0003, ANOVA, Tukey’s *post hoc*, Figure 4B). This increase in similarity was sustained until the end of the study, 8 weeks from FMT (Figure 4B). In contrast, in the Ab-lav-sham group the similarity to donors decreased from baseline due to the antibiotic treatment and did not recover in the follow-up time points at 2 weeks (*p* = 3.97 × 10^–4^, ANOVA, Tukey’s *post hoc*) and 8 weeks (*p* = 1.71 × 10^–4^, ANOVA, Tukey’s *post hoc*) after the sham FMT. In addition, the Ab-lav-sham group separated significantly from other treatment groups in PCoA analyses at the end of the study concerning both fecal (*p* = 0.001, PERMANOVA, Figure 4C and Supplementary Figure S1D) and mucosal (*p* = 0.001, PERMANOVA, Figure 4D and Supplementary Figure S2) microbiota composition. In summary, FMT restored the microbial diversity disrupted by antibiotics, but antibiotic pre-treatment did not increase the overall similarity between donor and recipient microbiota, as compared with control groups.

### Specific Genus-Level Differences in Microbial Composition After FMT Are Attributable to Antibiotic Pre-treatment

**FIGURE 5:**
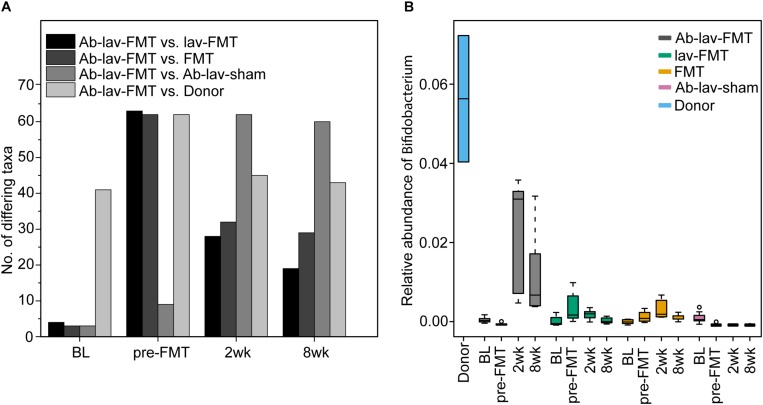
**(A)** Number of genus-level bacterial taxa that differed in relative abundance (FDR-adjusted *p* < 0.05) between the Ab-lav-FMT group versus all other groups, or versus donor, at four different time points. **(B)** Relative abundance of genus *Bifidobacterium*, demonstrating higher levels in Balb/c donor than in C57BL/6 recipient mice. Relative abundance increased in the Ab-lav-FMT group at 2 and 8 weeks after FMT compared with baseline (*p* = 5.59 × 10^–9^ and 6.02 × 10^–6^, respectively), and also compared with other groups at 2 weeks after FMT (FDR-adjusted *p* < 0.05). On the other hand, levels of *Bifidobacterium* were decreased in the Ab-lav-sham group after antibiotic treatment and remained low until 8 weeks after sham FMT.

To assess the magnitude of the changes introduced by the antibiotic pre-treatment, we determined the number of significantly differing taxa between the Ab-lav-FMT and other groups as well as the donors at different time points (Figure 5A). The obtained FDR-adjusted *p*-values were calculated using generalized linear mixed models. At baseline, the donors had the greatest number (41 of 117 detected) of differing taxa relative to the Ab-lav-FMT group, which is consistent with the mouse strain effect. After the antibiotic pre-treatment, the number of differing taxa increased sharply compared with the animals not treated with antibiotics. During the follow-up period the Ab-lav-FMT group remained relatively different from the other FMT-treated groups (19–32 of the genera showed a statistical difference), which remained highly similar to each other during the follow-up period (only 3–4 of the genera showed a statistical difference between lav-FMT and FMT groups, Supplementary Figures S3A–C). Throughout the study, the number of taxa differing between all recipient groups and the donors remained high (37–62 of the genera, Supplementary Figures S3A–D).

Next, we determined the number of genus-level taxa that were differentially engrafted in mice pre-treated with antibiotics compared with the control groups. This was done again by using a generalized linear mixed model to test the difference between study groups at both follow-up time points. Two weeks after FMT, there were 28 significantly different taxa between the Ab-lav-FMT and lav-FMT groups, and 32 between the Ab-lav-FMT and FMT groups. Antibiotic pre-treatment had a substantial effect on the engraftment since there were only three taxa with significantly different abundance 2 weeks after FMT between the lav-FMT and FMT groups (Supplementary Figures S3B,C). Thus, antibiotic pre-treatment had a detectable effect on the taxa level, but only a minor effect on overall microbiota engraftment after FMT.

To determine whether the antibiotic pre-treatment improved the engraftment of specific taxa, we compared the mean relative abundances of the significantly different taxa in different groups 2 weeks after FMT. Taxa that were detected in the donors but were absent in the Ab-lav-sham group and were more increased in group Ab-lav-FMT than in groups lav-FMT or FMT were deemed to have benefited in their engraftment from antibiotic pre-treatment. There were five genus-level taxa that matched these criteria: *Bifidobacterium, Adlercreutzia, Enterorhabdus, Odoribacter*, and *Alkalibacterium* (Supplementary Table S4). At baseline, the genus *Bifidobacterium* was present in recipient mice on average at 0.13% ± 0.001 abundance, and in donors at 5.72% ± 2.26 abundance (Figure 5B). Two weeks after FMT, the bifidobacterial abundance in the lav-FMT and FMT groups remained low (on average 0.34% ± 0.002), whereas in the Ab-lav-FMT group it increased significantly to the level of 2.25% ± 0.014 (*p* = 5.59 × 10^–9^). In this group, bifidobacterial abundance remained increased also at 8 weeks after FMT (1.29% ± 0.012, *p* = 6.02 × 10^–6^). Overall, the antibiotic pre-treatment had a detectable effect on the engraftment of specific genus-level taxa, most notably on the abundance of *Bifidobacterium*.

In a similar way, we sought bacterial taxa that were decreased after FMT in mice pre-treated with antibiotics, but were present in the other FMT groups on higher level 2 weeks after FMT-treatment. In total, eight genus-level taxa were significantly less abundant (FDR-adjusted *p*-value < 0.05; Supplementary Table S4) in Ab-lav-FMT group than in other FMT-treated groups. Thus, antibiotic pre-treatment suppressed some taxa that were not re-introduced by FMT from the other mice strain. On the other hand, antibiotic treatment as such increased the relative abundance of three genus-level taxa, and *Marvinbryantia*, *Stomatobaculum*, and an uncultured member of Firmicutes (*incertae sedis*) increased in both antibiotic-treated groups, i.e., groups with and without FMT (Supplementary Table S4). Interestingly, the abundance of *Verrucomicrobium* was increased after the antibiotic pre-treatment (Figure 2C), was not detected in the donors, and the abundance of this taxa was decreased in all groups after FMT, but not after sham FMT (Supplementary Table S4).

### Species Richness in Donor and Recipient Mice

At baseline, donors had significantly higher richness than recipient mice (*p* = 1.0 × 10^–7^, for all group comparisons, ANOVA, Tukey’s *post hoc*, Supplementary Figure S4). The higher richness was transferred with FMT to all treated animals, where the richness remained high at the follow-up time points (*p* < 0.001, ANOVA, Tukey’s *post hoc*, Supplementary Figure S4). Thus, significant difference in richness between Ab-lav-FMT and other FMT-treated groups was not detected 2 or 8 weeks after FMT-treatment (comparison of Ab-lav-FMT and lav-FMT 2 weeks after FMT-treatment *p* = 0.99, Ab-lav-FMT and FMT 2 weeks after FMT-treatment *p* = 0.98 and comparison of Ab-lav-FMT and lav-FMT eight weeks after FMT-treatment *p* = 0.27, Ab-lav-FMT and FMT 2 weeks after FMT-treatment *p* = 0.99, ANOVA, Tukey’s *post hoc*).

### Microbial Composition in Colonic Mucosa and Length of Colon

At the end of the study, the coecum microbiota of the FMT-treated mice was composed mainly of Firmicutes (89.50% ± 0.83) and Bacteroidetes (6.08% ± 0.50), whereas microbiota in the sham-FMT group was dominated by only Firmicutes (96.99%) (Supplementary Figure S5). Similarly, mainly Firmicutes and Bacteroidetes were detected in the distal colon of the FMT-treated animals (82.90% ± 4.51 and 11.93% ± 4.11, respectively), but in the sham-FMT group the microbiota was dominated by Firmicutes (68.61%) and Deferribacteres (29.03%) (Supplementary Figure S5).

In PCoA, mucosal samples from donors and all FMT-treated animals clustered together, whereas the Ab-lav-sham group separated from the others (*p* = 0.001, PERMANOVA, Figure 4D and Supplementary Figure S2). Moreover, similarity to donor microbiota was low for the Ab-lav-sham group (Supplementary Figure S6). In contrast, all of the FMT groups showed high similarity to donor microbiota (average ρ = 0.78 ± 0.03), but the highest similarity was observed in the Ab-lav-FMT group (ρ = 0.80 ± 0.02). Thus, colonic microbiota in the FMT groups resembled those of the donors, whereas the Ab-lav-sham group had clearly distinct colonic microbiota profiles.

**FIGURE 6:**
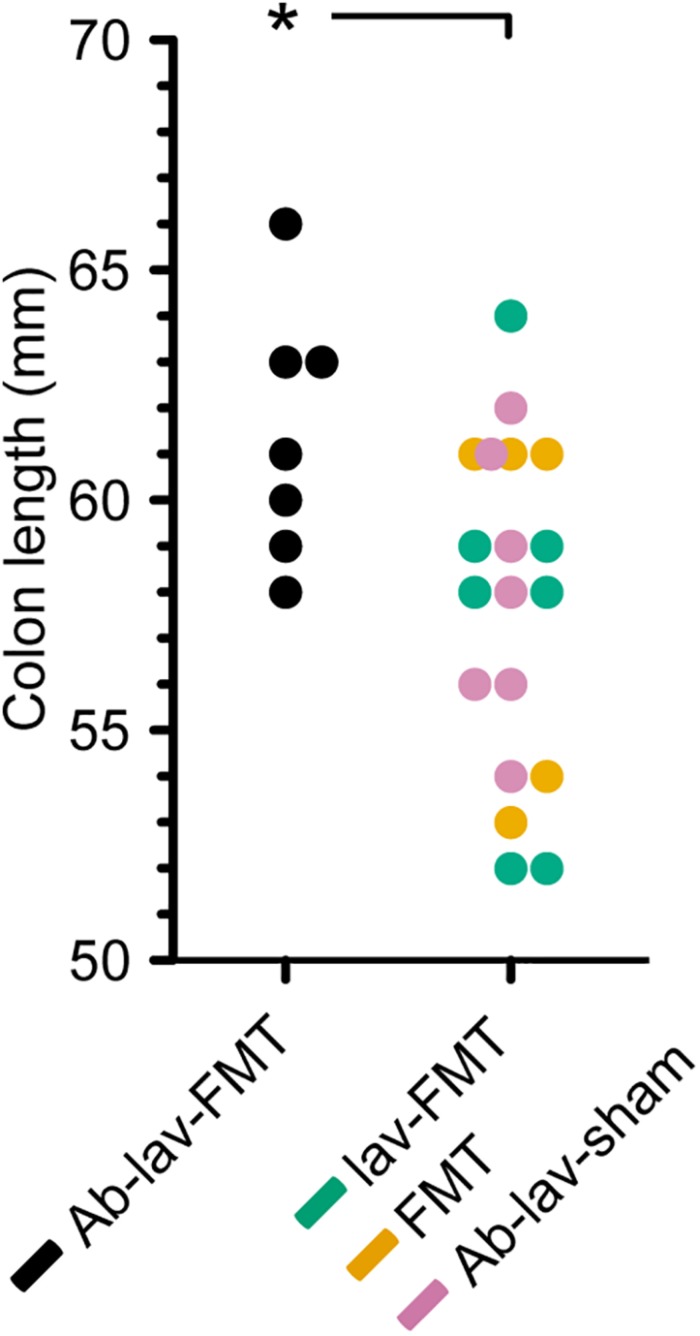
Colon length in the four treatment groups (C57BL/6 recipient mice). Significance level indicated with an asterisk = *p* < 0.05.

Colon length is known to differ between different mouse strains and/or colonies, and previous studies have demonstrated that Balb/c mice have greater colon length than C57BL/6 mice (Qualls et al., 2006). A comparison of colon length in the four C57BL/6 recipient groups showed that mice treated with antibiotics, lavage, and FMT had significantly longer colons than mice from all other groups combined, resembling the reported Balb/c donor phenotype (Figure 6, *p* = 0.02, Student’s *t*-test).

## Discussion

In this study, we assessed the effect of antibiotic pre-treatment on the engraftment of donor microbiota in FMT. Balb/c mice were used as FMT donors and C57BL/6 mice as the FMT recipient strain. At baseline, microbiota composition and richness differed between donors and recipients. As expected, pre-treatment with ciprofloxacin, metronidazole, and vancomycin significantly decreased the bacterial diversity. After FMT, the diversity, species richness, and similarity to donor microbiota rose in the Ab-treated group, but the same levels were also reached in the other FMT groups. Nonetheless, we found effects of the antibiotic pre-treatment on the engraftment of specific donor microbiota to recipient mice. Interestingly, abundance of a number of genus-level taxa, including *Bifidobacterium*, was significantly higher in the Ab-treated group 2 weeks after FMT than in the other study groups. Moreover, colon length in the Ab-treated group was significantly longer than in other recipient mice.

Our results demonstrating differences at baseline between two mouse strains are in line with those observed in an earlier metagenomic study, which examined five factors, including mouse strain and provider, affecting the composition of the mouse gut microbiome (Xiao et al., 2015). In another study, higher richness of donor stool relative to recipient stool was seen as a positive predictor of microbiota engraftment after FMT (Ericsson et al., 2017). In our study, donors had significantly higher richness than recipients, indicating that conditions for donor microbiota engraftment were favorable. Overall, the results suggested that higher species richness was transferred to FMT recipients, but antibiotic pre-treatment offered no significant benefit.

The antibiotic cocktail (ciprofloxacin, metronidazole, and vancomycin) used in this study was active against a broad spectrum of Gram-negative, Gram-positive, and anaerobic bacteria and was regarded as suitable for clinical application. As expected, the pre-treatment decreased diversity and reduced the number of detected taxa. These findings are consistent with those of Dethlefsen and Relman (2011), who reported a shift in the distal gut microbial composition and a decrease of diversity after two courses of ciprofloxacin. In the current study, at genus level, taxa belonging to *Verrucomicrobium*, *Lactobacillus*, and *Parabacteroides* dominated the composition in the recipient mice after pre-treatment with antibiotics. The genera *Lactobacillus* and *Parabacteroides* were detected in both groups treated with antibiotics until the end of the study, whereas the abundance of *Verrucomicrobium* decreased after FMT in animals treated with antibiotics. Many *Lactobacillus* spp. are known to harbor resistance genes to vancomycin and ciprofloxacin or to have a resistant phenotype (Campedelli et al., 2019), and members of the *Bacteroides fragilis* group, including *Parabacteroides distasonis*, are among the anaerobes least susceptible to antibiotics (Schuetz, 2014). Therefore, it is not surprising that *Lactobacillus* and *Parabacteroides* were found to prevail after the antibiotic treatment. Verrucomicrobia, on the other hand, are a special phylum of bacteria that have complex endomembrane structures and a compartmentalized cell plan (Lee et al., 2009), which may account for their resistance against antibiotics. While we are unaware of the traits that conferred resistance of *Verrucomicrobium* to the antibiotics applied, its high abundance in Ab-treated animals indicates effective mechanisms. Notably, antibiotic resistance of specific bacteria by deactivation mechanism may give collective resistance also to other members of the microbial community (Sorg et al., 2016). Indeed, in addition to the above-mentioned three taxa, a number of other bacteria were also present in low amounts in the Ab-treated mice, and these may represent such co-benefiting taxa.

Although in our study the FMT restored microbial diversity after antibiotic pre-treatment, the results did not show a significant increase in overall similarity to donor microbial composition compared with animals not treated with antibiotics. Furthermore, the similarity analyses showed a higher correlation of the microbial composition in mice from all FMT groups with their own baseline, rather than with the donor communities, indicating resilience of the host microbiota. Therefore, the results of this study of FMT between two different inbred strains of mice did not support the main hypothesis that antibiotic pre-conditioning will lead to a more effective transfer of donor microbiota to the recipient as a whole. Nevertheless, specific taxa were shown to benefit from the antibiotic pre-treatment.

Here we showed that there were detectable effects on the abundance of specific bacterial taxa after the FMT. Five presumably Balb/c donor-derived genus-level taxa, including *Bifidobacterium*, were more abundant in the stool of recipients that had been pre-treated with antibiotics. In this group, bifidobacterial abundance remained increased until the end of the study. Since bifidobacteria are non-pathogenic, generally regarded as safe, and also used as probiotics, their promotion by antibiotic pre-treatment may be of interest for FMT, particularly as they are also known to exert anti-inflammatory and epithelium-reinforcing actions (Hiippala et al., 2018). Interestingly, a recent study in humans reported that *Bifidobacterium* was more abundant after FMT following pre-treatment with ciprofloxacin, although this was not compared with FMT without antibiotic pre-treatment (Kang et al., 2017). Furthermore, the colon length of mice pre-treated with antibiotics before FMT was increased at the end of the study relative to all other groups, indicating that the differences in microbiota composition may have had physiological effects, affecting the mouse phenotype.

Previous studies have produced divergent results regarding the effects of bowel cleansing prior to microbiota engraftment. One study demonstrated a positive effect of bowel cleansing (Le Roy et al., 2018), while another study found no impact of lavage alone, but a combined effect with lavage and antibiotics (Ji et al., 2017). While our study primarily addressed the effects of antibiotic pre-treatment on donor microbiota engraftment, group comparisons suggested no improvement of engraftment by bowel lavage versus no bowel lavage. However, these results should be interpreted with caution because we had chosen a lower laxative dose for mice than the studies mentioned above. In humans, there is evidence that the effect on microbial composition is larger when higher amounts of laxative are used (Jalanka et al., 2015). Also, a recent rodent study showed that a high dose of purging agent introduced a significant reduction in bacterial load, resulting in better microbial engraftment (Wrzosek et al., 2018). Thus, an efficient lavage could provide some advantage for FMT, and attention should be paid to the lavage regimes in future studies.

In summary, we showed that in FMT between two different mice strains, mimicking the clinical settings, pre-treating the mice with broad-spectrum antibiotics had only a minor effect on donor microbiota engraftment. One of the observations was better engraftment of the genus *Bifidobacterium* with antibiotic pre-treatment, which may offer potential benefits in future therapeutic studies. This was supported by the finding that colon length was greater in animals pre-treated with antibiotics, thereby mimicking the donor strain. Since the resilience of the recipient’s microbiota appeared to limit the engraftment, a potential use for antibiotics before FMT may be the modulation of recipient microbiota profiles. Recipient microbiota modulation by antibiotic pre-treatment may act synergistically with donor-derived microbiota in resolving dysbiosis in human diseases.

## Data Availability Statement

The raw sequencing data for this study can be found in the European Nucleotide Archive (PRJEB33286).

## Ethics Statement

The animal study was reviewed and approved by the Southern Finnish State Administrative Agency (ESAVI/1286/04.10.07/2016).

## Author Contributions

TF, SM, V-JA, EM, PA, and RS conceived and designed the study. JJ contributed to conception and design of the study. TF, JJ, AH, HJ, and CS performed animal work and laboratory analysis. AH and JJ performed the statistical analysis. TF, AH, JJ, and RS interpreted the results and wrote the first draft of the manuscript. All authors contributed to manuscript revision and read and approved the submitted version.

## Conflict of Interest

The authors declare that the research was conducted in the absence of any commercial or financial relationships that could be construed as a potential conflict of interest.

## Abbreviations

Ab: antibiotic
ANOVA: analysis of variance
BL: baseline
FMT: fecal microbiota transplantation
IBS: irritable bowel syndrome
Lav: lavage
PBS: phosphate-buffered saline
PCoA: principal coordinates analysis
PERMANOVA: permutational multivariate analysis of variance
rCDI: recurrent *Clostridioides difficile* infection
UC: ulcerative colitis.
