# Supplementary material for: Minor Effect of Antibiotic Pre-treatment on the Engraftment of Donor Microbiota in Fecal Transplantation in Mice
Source: Front Microbiol. 2019 Nov 21;10:2685. doi: 10.3389/fmicb.2019.02685 (PMC6881239; doi:10.3389/fmicb.2019.02685)
Supplement: Supplementary file 2 [file Data_Sheet_1.PDF]

Supplementary Material

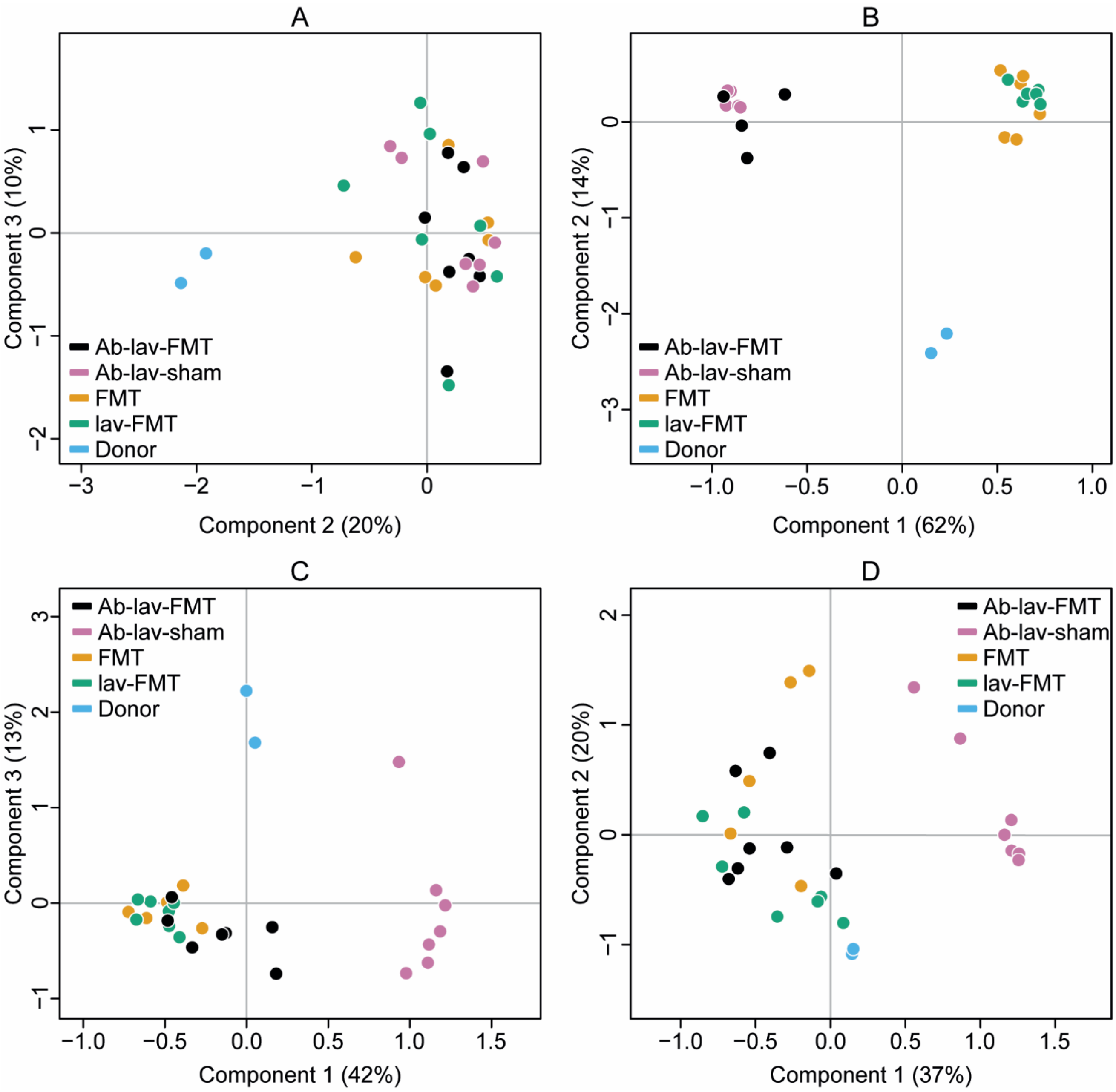

Supplementary Figure 1. (A) PCoA of fecal microbiota composition based on OTU-level at baseline. **(B)** PCoA of fecal microbiota composition based on OTU-level at time point pre-FMT. **(C)** PCoA of fecal microbiota composition based on OTU-level 2 weeks after FMT. **(D)** PCoA of fecal microbiota composition based on OTU-level 8 weeks after FMT.

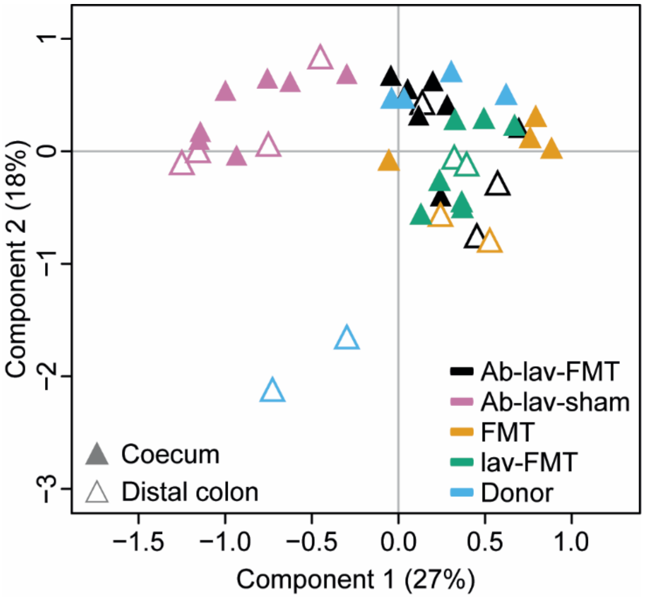

Supplementary Figure 2. PCoA of mucosal microbiota composition based on OTU-level 8 weeks after (sham) FMT.

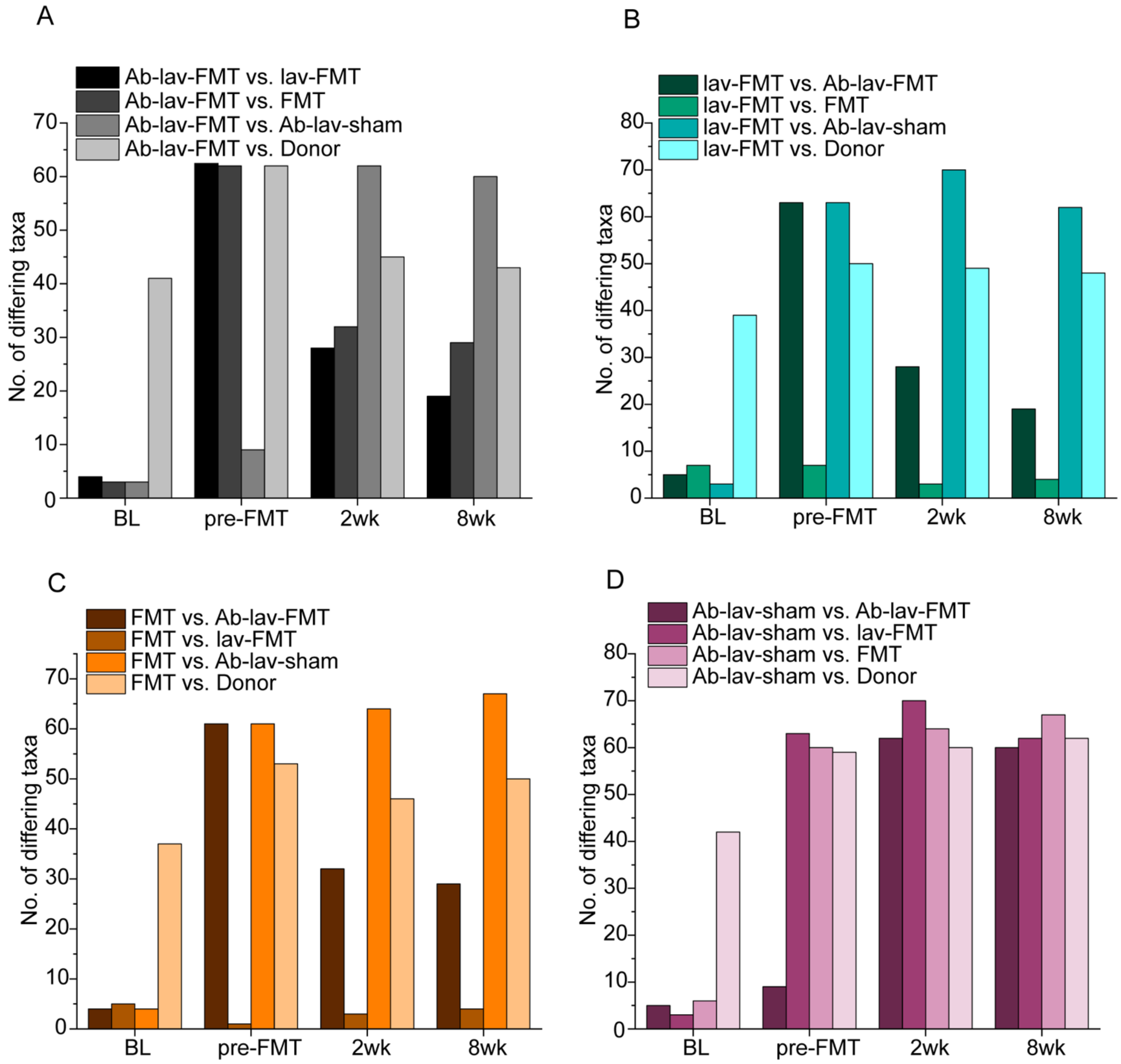

Supplementary Figure 3. The number of significant FDR-adjusted p-values (FDR-adjusted $p<0.05$) indicating the number of differing genus level taxa. The obtained FDR-adjusted p-values were calculated using generalized linear mixed models. All study groups are compared to each other across all time points. **(A)** Ab-lav-FMT versus other groups. **(B)** lav-FMT versus other groups. **(C)** FMT versus other groups. **(D)** Ab-lav-sham versus other groups.

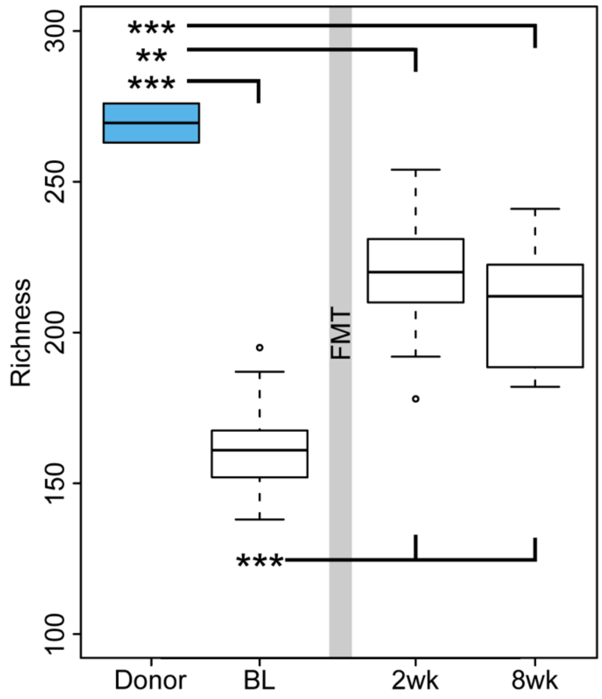

Supplementary Figure 4. Species richness in Balb/c donor and C57BL/6 recipient mice at baseline (BL), and richness in all FMT-treated groups combined 2 and 8 weeks after FMT treatment. Significance level indicated with asterisks: ** = $p<0.01$, *** = $p<0.001$, ANOVA, Tukey's post hoc.

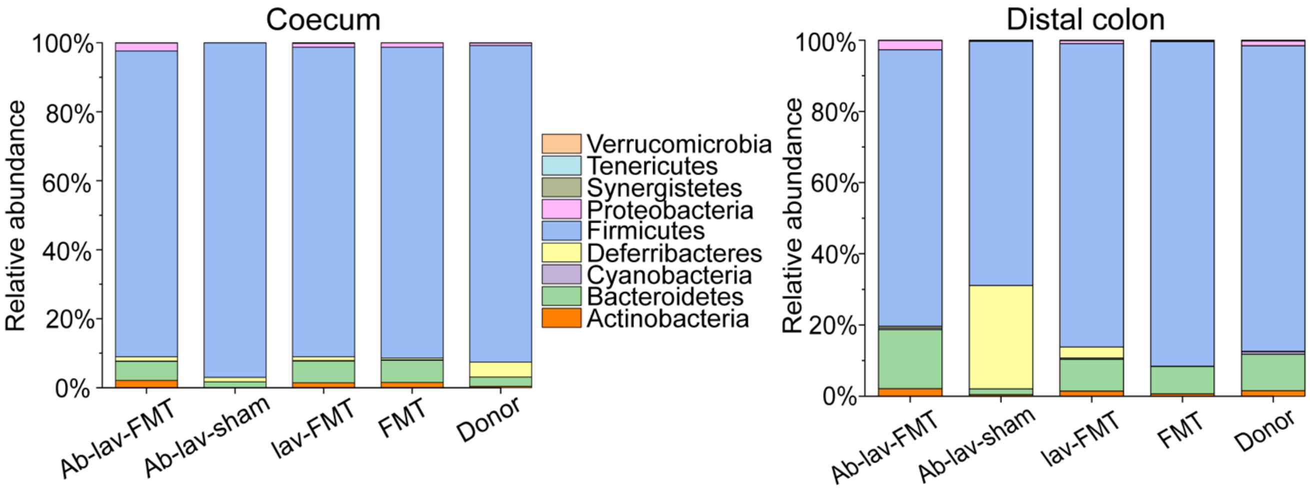

Supplementary Figure 5. Microbiota composition in mucosal samples in coecum and distal colon at the end of the study (week 8).

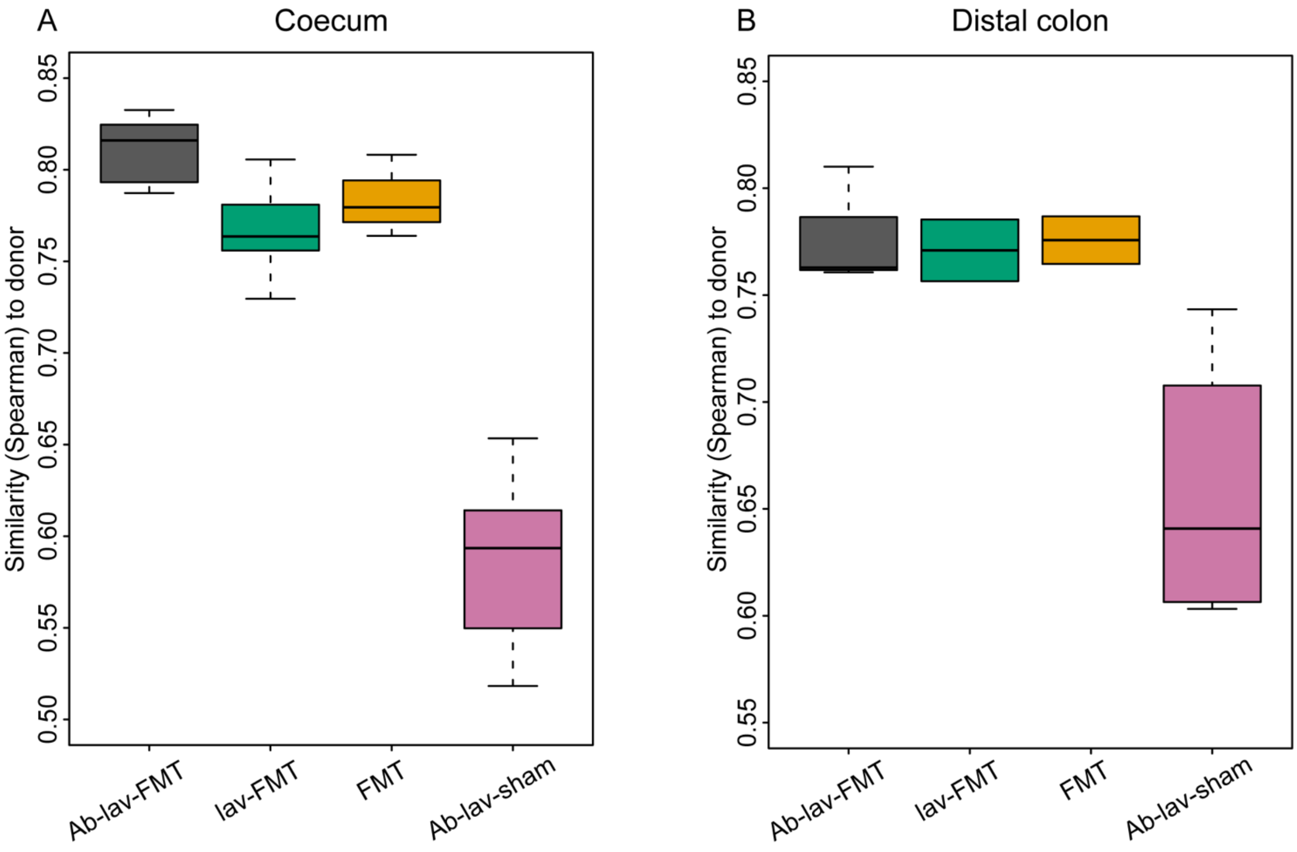

Supplementary Figure 6. Average Spearman correlation of mucosal microbiota to donor in all study groups. **(A)** Location coecum. **(B)** Location distal colon.
